# Modeling radiologists’ cognitive processes using a digital gaze twin to enhance radiology training

**DOI:** 10.1038/s41598-025-97935-y

**Published:** 2025-04-21

**Authors:** Akash Awasthi, Anh Mai Vu, Ngan Le, Zhigang Deng, Supratik Maulik, Rishi Agrawal, Carol C. Wu, Hien Van Nguyen

**Affiliations:** 1 Department of Electrical and Computer Engineering, University of Houston, Houston, USA; 2 Department of Computer Science & Computer Engineering, University of Arkansas, Fayetteville, USA; 3 Department of Computer Science, University of Houston, Houston, TX USA; 4 Triradiate Industries, Sugarland, TX USA; 5 Department of Thoracic Imaging, Division of Diagnostic Imaging, The University of Texas MD Anderson Cancer Center, Houston, TX USA; 6 Department of Electrical and Computer Engineering, University of Houston, Room no- N368, Cullen College of Engineering Building-14222 Martin Luther King Blvd, Houston, TX USA

**Keywords:** MedGaze, Scanpath prediction, Large multimodal Models(LMMs), Computational science, Biomedical engineering, Radiography

## Abstract

Predicting human gaze behavior is critical for advancing interactive systems and improving diagnostic accuracy in medical imaging. We present MedGaze, a novel system inspired by the “Digital Gaze Twin” concept, which models radiologists’ cognitive processes and predicts scanpaths in chest X-ray (CXR) images. Using a two-stage training approach—Vision to Radiology Report Learning (VR2) and Vision-Language Cognition Learning (VLC)—MedGaze combines visual features with radiology reports, leveraging large datasets like MIMIC to replicate radiologists’ visual search patterns. MedGaze outperformed state-of-the-art methods on the EGD-CXR and REFLACX datasets, achieving IoU scores of 0.41 [95% CI 0.40, 0.42] vs. 0.27 [95% CI 0.26, 0.28], Correlation Coefficient (CC) of 0.50 [95% CI 0.48, 0.51] vs. 0.37 [95% CI 0.36, 0.41], and Multimatch scores of 0.80 [95% CI 0.79, 0.81] vs. 0.71 [95% CI 0.70, 0.71], with similar improvements on REFLACX. It also demonstrated its ability to assess clinical workload through fixation duration, showing a significant Spearman rank correlation of 0.65 (*p* < 0.001) with true clinical workload ranks on EGD-CXR. The human evaluation revealed that 13 out of 20 predicted scanpaths closely resembled expert patterns, with 18 out of 20 covering 60–80% of key regions. MedGaze’s ability to minimize redundancy and emulate expert gaze behavior enhances training and diagnostics, offering valuable insights into radiologist decision-making and improving clinical outcomes.

**Supplementary Information:**

The online version contains supplementary material available at 10.1038/s41598-025-97935-y.

## Introduction

The modeling of human gaze behavior is a critical problem in computer vision, with significant implications for designing interactive systems that can anticipate the user’s attention. In medical imaging, particularly with chest X-rays (CXR), predicting scanpaths plays a pivotal role in enhancing diagnostic accuracy and efficiency^1,2^. Scanpath prediction not only helps in understanding how expert radiologists visually navigate these images to detect abnormalities but also provides a window into their cognitive processes during diagnosis^3^. This is crucial for modeling how they prioritize, interpret, and synthesize visual information^4,5^. By analyzing expert radiologists’ gaze behavior, we can develop advanced training programs that guide novice radiologists to adopt effective viewing strategies, reducing diagnostic errors and improving clinical skills^6^.

A key aspect of our work is the concept of the Digital Gaze Twin, a virtual representation that mimics how an expert radiologist views medical images. This system enables novice radiologists to follow expert gaze patterns, optimizing their visual trajectories when diagnosing CXR images. The Digital Gaze Twin aligns with Eye Movement Modeling Examples (EMMEs), a well-established concept in educational psychology and medical education. EMMEs are instructional tools that visually demonstrate expert gaze patterns to learners, helping them adopt effective visual search strategies and improve cognitive processing during complex tasks^7,8^. In medical education, EMMEs have been shown to enhance diagnostic accuracy by guiding novices to focus on clinically relevant regions and prioritize information in a manner similar to experts^9,10^.

However, traditional EMME approaches face several limitations. For instance, they often rely on static, pre-recorded gaze patterns from a limited set of cases, which may not adapt to individual learner needs or scale across diverse medical scenarios^11,12^. Additionally, traditional EMMEs may simplify gaze patterns due to technical constraints, potentially omitting critical aspects of expert visual search strategies, such as fixation orders, dwell times, and transitions between regions^13^. The Digital Gaze Twin addresses these limitations by leveraging advanced AI techniques to create a dynamic and scalable virtual representation of expert gaze behavior. Unlike static EMMEs, the Digital Gaze Twin generates real-time, context-aware scanpaths tailored to specific radiology reports and abnormalities. This adaptability ensures that novice radiologists are exposed to a wide range of diagnostic scenarios, enhancing their ability to generalize learned strategies to new cases.

**Fig. 1:**
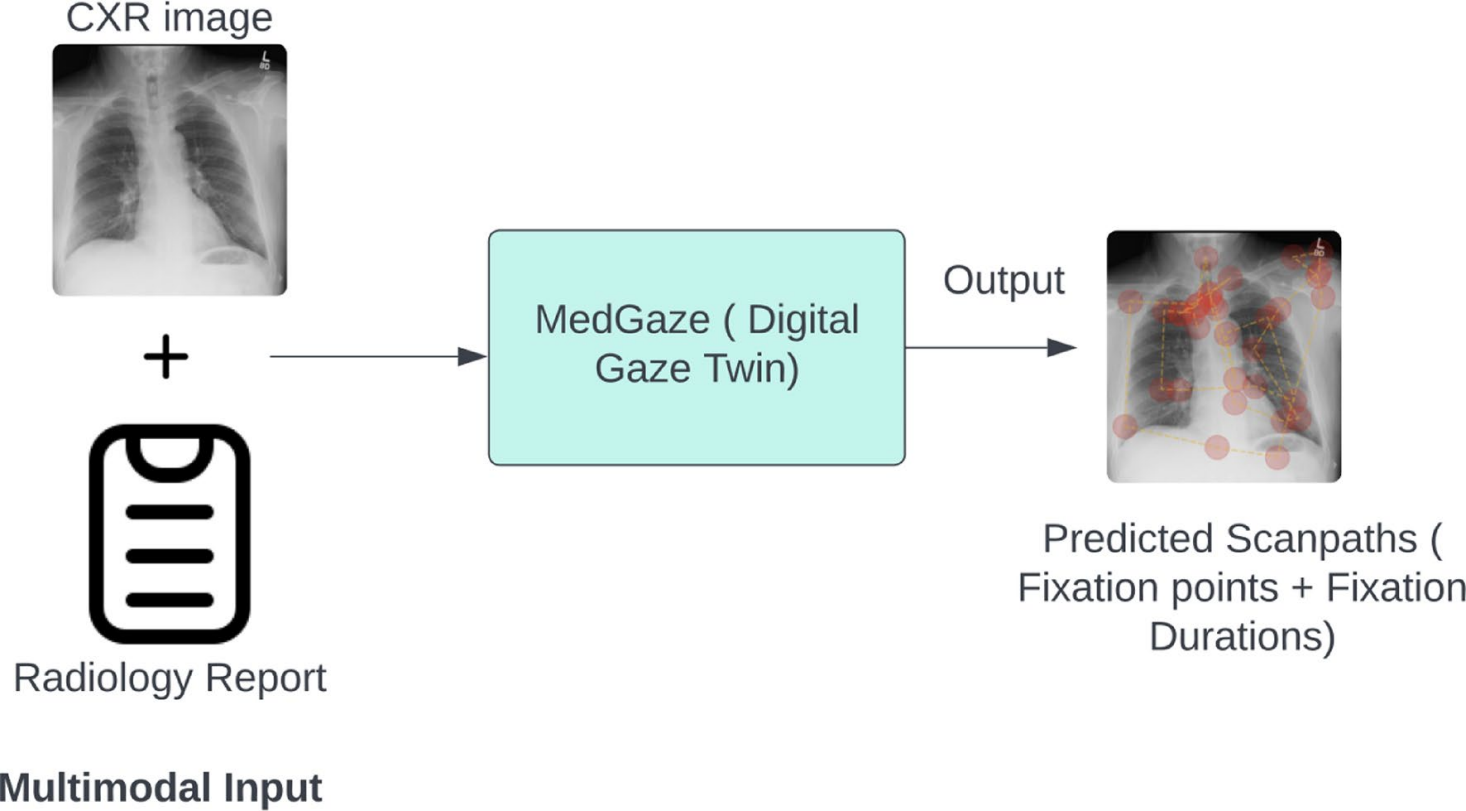
Overview of MedGaze—A Digital Gaze Twin utilizing LMMs. This system integrates multimodal inputs, such as CXR images and radiology reports, to predict fixation points and their durations, highlighting critical regions in the images. The output shows predicted fixation points as red dots, with dot sizes scaled by predicted fixation durations to represent the attention levels required for different areas. MedGaze can potentially serve as an educational tool for radiology residents and medical students, guiding them in identifying key areas and teaching effective viewing strategies for CXR image interpretation, ultimately enhancing diagnostic accuracy.

Predicting human scanpaths on medical images presents unique challenges compared to natural images due to the presence of abnormal regions with varying shapes, sizes, and contrasts^14^. Previous research has focused on predicting scanpaths in natural images by targeting specific objects or goals^15–17^. Our study introduces MedGaze (shown in Fig. 1), a novel system tailored to model scanpaths aligned with radiology reports containing multiple abnormalities. MedGaze predicts fixation points and durations crucial for identifying abnormalities, aiming to enhance human-AI collaboration and refine training modules for novice radiologists. By simulating expert gaze patterns, MedGaze serves as the foundation for a training system that helps learner develop more effective visual search strategies for diagnosing abnormalities.

**Fig. 2:**
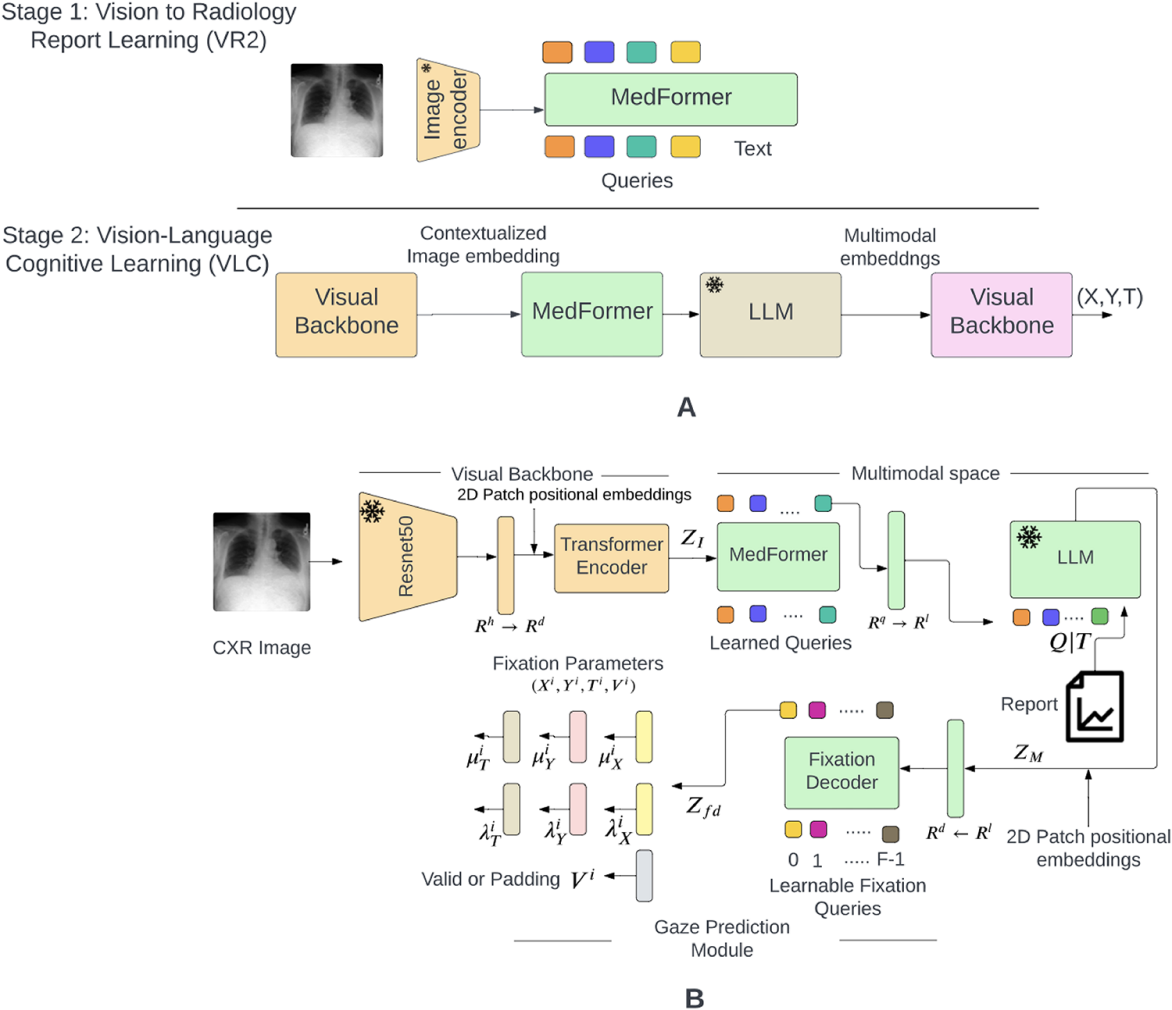
Representation of the proposed methodology. Subfigure (**A**) illustrates the two-stage training strategy: VR2 followed by VLC. This approach is designed to extract medically relevant multimodal features and model the cognitive processes of radiologists during CXR image diagnosis. Subfigure (**B**) depicts the architecture of the proposed model MedGaze, emphasizing the integration of the VLC stage.

As shown in Fig. 2a, our methodology involves two-stage training: Vision to Radiology Report Learning (VR2) and Vision Language Cognitive Learning (VLC), utilizing large publicly available datasets. Given the limited availability of eye gaze tracking data^1,2^, we leverage the MIMIC dataset^18,19^ for representation learning to extract medically relevant multimodal features, which are then used to model eye gaze movements. Our model employs Large Multimodal Models (LMMs) to extract text-enriched multimodal embeddings. Unlike previous computer vision efforts that focus on predicting scanpaths based on specific objects or categories, our approach addresses a broader context of modeling scanpath sequences for searching multiple abnormalities in CXR images. Specifically, the key technical innovation of MedGaze is its capability to model fixation sequences that are an order of magnitude longer than those handled by the current state-of-the-art methods.

To validate our approach, we compare it to current state-of-the-art methods in computer vision for predicting scanpaths on natural images, using statistical metrics. Additionally, we assess our model’s ability to generalize across different radiologists. An expert thoracic radiologist provides ratings based on the comprehensiveness and redundancy of predicted scanpaths to evaluate their clinical relevance. We also present an innovative application of our model in identifying the most challenging or time-consuming cases within a dataset, aiding in the prioritization and efficient management of diagnostic workflows.

The main contributions of our work are as follows:

- Introduction of the Digital Gaze Twin: A virtual model mimicking expert radiologists’ scanpaths, aiding novice radiologists in adopting more effective searching strategies and improving diagnostic accuracy. This concept extends and overcomes limitations of traditional EMMEs by providing a dynamic and scalable framework for modeling expert gaze behavior.
- Development of MedGaze: A novel AI system designed to predict scanpaths and fixation durations in chest X-rays, addressing the unique challenges of medical imaging.
- Two-stage training approach: Combines Vision to Radiology Report Learning (VR2) and Vision Language Cognitive Learning (VLC) for multimodal feature extraction using large-scale datasets.
- Advanced modeling of complex fixation sequences on detailed radiology reports: MedGaze predicts longer and more intricate scanpaths based on detailed radiology reports, unlike prior methods focused on specific objects^15–17^, offering a more realistic representation of radiologists’ eye movements across complex medical images.
- Comprehensive evaluation: Performance validation through statistical metrics, expert radiologist evaluation, and demonstration of MedGaze’s clinical relevance by identifying challenging cases and managing diagnostic workflow.

## Materials and methods

Our methodology, as illustrated in Fig. 2a, employs a two-stage training approach to model the complex cognitive processes involved in diagnosing CXR images. To address the challenge of limited eye gaze tracking data^1,2^, we begin with the VR2 phase, which focuses on extracting and enhancing visual features from CXR images. This phase is followed by the VLC phase, which integrates these visual features with radiology reports to create comprehensive multimodal embeddings and predict scanpaths. Inspired by BLIP2^20^, this two-stage process ensures that our model effectively captures both visual and textual information, resulting in precise gaze pattern predictions and improved diagnostic reasoning.

Figure 2a outlines our two-stage training approach, VR2 followed by VLC, aimed at extracting the text-enriched multimodal embeddings to model cognitive processes in CXR diagnosis. Figure 2b expands on our architectural framework of MedGaze in the VLC stage of training, which comprises three pivotal components: the Visual Backbone, the Multimodal Space, and the Gaze Prediction Module, as described below.

### Visual backbone

The Visual Backbone is essential for extracting contextualized visual embeddings. It includes ResNet-50^21^ as a frozen feature extractor that extracts visual features from images. Following this, six standard transformer encoder^22^ layers are incorporated to generate a contextualized feature embedding, denoted as $\:{Z}_{I}$. Additionally, we employ 2D sinusoidal positional embeddings to denote the location of each patch^23^. Our ablation study experiments reveal that substituting the ResNet-50 (feature extractor) and transformer encoder block with a CLIP-based vision^24^ transformer results in increased training time and computational costs, as well as relatively inferior performance. We include the ablation study results in the supplementary material.

### MedFormer

During the initial training phase of the VR2, we propose a transformer-based module pre-trained specifically on the MIMIC data, called MedFormer. This module aims to bridge the gap between the frozen image encoder and the large language model, facilitating the extraction of a fixed number of image features irrespective of the input image resolution. It consists of two transformer submodules: one called an image transformer which interacts with the frozen image encoder, and the other one called a text transformer which can function both as a text encoder and decoder. MedFormer filters out unnecessary visual details, providing focused and refined visual context. This reduces the LLM’s burden of aligning visual and language data from scratch, making the training process more efficient.

### Large language model

This component serves as the cornerstone of our architecture, tasked with modeling the complex interplay between refined contextualized image embeddings and text embeddings. Consequently, it equips the gaze prediction module with robust multimodal embeddings enriched by textual context. By employing the frozen decoder-based LLM known as OPT^25^, we integrate MedFormer’s output with text embeddings and input this concatenated representation to the LLM. Medformer’s output takes the form of *batch size × number of queries × query dimension (32 × 32 × 768)*, which is different from the text embeddings of the OPT model. To reconcile this discrepancy, we introduce a linear layer to project the hidden dimension of Medformer’s output to align with the hidden dimension of the OPT model, which is set at 2048. Afterward, we concatenate these representations, adding padding to the maximum text length, usually 32, to prevent size mismatch. Therefore, the input to the OPT model is shaped as *batch size x number of queries + padding (max length) x OPT model dimension*. During the Vision Language Cognition (VLC) stage of training, the LLM remains unchanged.

### Multimodal space

In contrast to the previous Gazeformer^15^ model, which employs simple linear projections to create image-text joint embeddings in the visual-semantic space, our experiments demonstrate that this approach falls short for detailed radiology reports. Therefore, we propose to connect MedGaze with a large language model to capture the complex interplay between image and text embeddings. Radiology reports are extensive, detailing numerous diseases or abnormalities that radiologists look for. Thus, simplistic modeling within the visual-semantic space may prove inadequate. The radiology reports show long dependencies since they begin searching for various diseases from the start of the image. Consequently, the sequence in which diseases are identified may involve complex cognitive processes. For example, detecting a patchy opacity of various sizes and shapes could lead to the diagnosis of pneumonia or edema. Our ablation experiments found that the optimal configuration for multimodal space requires integrating both MedGaze and the LLM.

### Gaze prediction module

This module is responsible for predicting both fixation coordinates and fixation duration, and it consists of a fixation decoder and a scanpath prediction network. Specifically, the fixation decoder adopts a transformer decoder-like architecture^22^, processing F fixation queries. These learnable fixation queries, which are randomly initialized, encode information about the fixation timestep. The maximum length of the fixation sequence is denoted as F. If the output fixation length is shorter than the maximum sequence length, padding is used to adjust the length to F. We have used six standard Transformer decoder layers in the Fixation decoder block. The latent fixation embeddings interact through self-attention and engage with the multimodal embedding (M) via encoder-decoder attention. Furthermore, fixed 2D position encoding is added to the multimodal embedding to provide positional information about the patches.

In the fixation prediction module, fixation coordinates are directly regressed from the output of the fixation decoder $\:{Z}_{fd}$, which has a size of $\:batch\:size\:\times\:$ F $\:\times\:\:model\:dimensions$, with F indicating the number of time steps. Radiologists exhibit variability in gaze sequence patterns, reflecting individual approaches while diagnosing CXR images, leading to inter-subject variability in fixation patterns. To ensure the model’s generalizability across multiple radiologists and avoid learning spurious correlations, fixation coordinates and durations are modeled using a Gaussian distribution.

The model regresses both the mean (µ) and log-variance (λ) of the 2D coordinates (X, Y) and fixation duration (T), which serve as the parameters of the Gaussian distribution that models fixation variability. Here, *X* = {*x*_*i*_}, Y = {y_i_​}, and T = {t_i​_} denote the sets of all fixation coordinates and durations across all time steps, where x_i_, y_i_, and t_i_ ​ represent the respective values at each time step. These parameters are learned through a multi-layer perceptron (MLP) network, where each layer in the MLP models a transformation of the input embeddings to estimate the parameters at each time step. The MLP layers output the mean and log-variance for each fixation coordinate (x, y) and duration (t) for each fixation time step, with separate MLP layers used for the x and y coordinates and for the duration. The mean (µ) represents the expected position or duration of the fixation, while the log-variance (λ) models the uncertainty or variability in these values across different subjects. To ensure the output is valid and remains within a reasonable range, the log-variance is exponentiated to produce the actual variance, making it always positive.

To allow for backpropagation through the stochastic process of sampling from a Gaussian distribution, we use the reparameterization trick^15,26^. This technique allows the model to generate samples from the Gaussian distribution while keeping the network differentiable. Specifically, the fixation coordinates and duration are generated by:

$$\:{X}_{i}=\:{\mu\:}_{\left\{{x}_{i}\right\}}+\:{\epsilon}_{\left\{{x}_{i}\right\}}\cdot\:exp\left(0.5\:{\lambda\:}_{\left\{{x}_{i}\right\}}\right)$$

$$\:{Y}_{i}=\:{\mu\:}_{\left\{{y}_{i}\right\}}+\:{\epsilon}_{\left\{{y}_{i}\right\}}\cdot\:exp\left(0.5\:{\lambda\:}_{\left\{{y}_{i}\right\}}\right)$$

$$\:{T}_{i}=\:{\mu\:}_{\left\{{t}_{i}\right\}}+\:{\epsilon}_{\left\{{t}_{i}\right\}}\cdot\:exp\left(0.5\:{\lambda\:}_{\left\{{t}_{i}\right\}}\right)$$

where $\:{\epsilon}_{\left\{{x}_{i}\right\}},{\epsilon}_{\left\{{y}_{i}\right\}},{\epsilon}_{\left\{{t}_{i}\right\}}$ are sampled from a standard normal distribution N(0,1) for each fixation time step. This allows us to sample different fixation coordinates and durations at each timestep while still allowing the network to be trained end-to-end via standard backpropagation.

Padding is employed for fixation sequences shorter than the maximum length set (F), and a separate MLP classifier with a softmax classifier is utilized to predict whether a specific step in the F slices of the multimodal embedding is a valid fixation or a padding token. During inference, (X, Y, T, V) are predicted, where X and Y represent the fixation coordinates, T represents the fixation duration, and V represents the probability of this fixation quad being a valid fixation or a padding token. Sequence termination occurs when V > 0.5, signaling the start of the padding tokens.

### Training procedure

In the initial phase (VR2), we train the MedGaze on the MIMIC data to acquire text-informed visual representation. During this stage, the Qformer^20^ is connected with the frozen image encoder to facilitate training using techniques such as Image-Text matching loss^27^, Image-Text contrastive loss^20^, and Image-Text grounding loss^28^. Moving to the second training stage (VLC), depicted in Fig. 2a, we integrate the MedGaze with the visual backbone (consisting of a frozen image encoder and a transformer encoder ) and the frozen LLM to execute the Vision-Language Cognitive Learning. In this phase, the total loss ($\:{L}_{t}$) is calculated by summing the spatio-temporal loss and the cross-entropy loss for token classification across N samples in the minibatch, as described in Eq. 1.

1 $$\:{L}_{t}=\frac{1}{N}{\sum\:}_{k=1}^{N}({L}_{spa}^{k}\:+\:{L}_{val}^{k}){}_{}^{}{}_{}^{}$$

Where $$L_{{spa}}^{k} = \frac{1}{{l^{k} }}\sum\nolimits_{{i = 0}}^{{l^{k} - 1}} {\left( {\left| {x_{i}^{k} \: - \hat{x}_{i}^{k} } \right| + \left| {y_{i}^{k} \: - \hat{y}_{i}^{k} } \right| + \left| {t_{i}^{k} \: - \hat{t}_{i}^{k} } \right|} \right)} \:$$

$$\:L_{{val}}^{k} = - \frac{1}{L}\sum\nolimits_{{i = 1}}^{{L - 1}} {\left( {v_{i}^{k} \:log\:\hat{v}_{i}^{k} + \left( {1 - v_{i}^{k} } \right)log\left( {1 - \hat{v}_{i}^{k} } \right)} \right)}$$

Here, $\:{L}_{t}$ represents the total loss, $\:{L}_{spa}$ is the spatio-temporal loss, which is an L1 loss between the predicted and ground truth fixation sequences, including duration. The predicted scanpath, denoted as $\:{s}^{k}=\left\{\right({x}_{i}^{k},{y}_{i}^{k},{t}_{i}^{k}){\}}_{i=0}^{L-1}\:$has a maximum length L, while $\:{l}_{l}^{k}$ is the length of the ground truth scanpath $\: \hat{s}^{k} =\left\{\right({x}_{i}^{k},{y}_{i}^{k},{t}_{i}^{k}){\}}_{i=0}^{{l}_{}^{k}-1}$. $\:{L}_{val}^{k}$ signifies the validity prediction loss, calculated as the negative log-likelihood loss for validity prediction for each token.

For the VLC training phase, we adopted a batch size of 32 and implemented Disjoint Optimization^15^ with the Adam optimizer^29^. This optimization technique employs variable learning rates for different network parameter groups. MedGaze underwent training for 200 epochs to achieve optimal performance.

### Datasets

In this study, we utilized two datasets: EGD-CXR^2^ and REFLACX^1^. These datasets consist of CXR images with synchronized eye-tracking and transcription pairs, annotated by different radiologists. We utilized both datasets to assess the generalization capability of our proposed system. Additionally, we merged both datasets to create a larger dataset, enabling us to evaluate the system’s performance comprehensively. Table 1 presents details about the training and testing samples utilized across different datasets. The key hyperparameter we considered was the maximum fixation length, set to 50. This choice was made based on the observation that most cases had a total of 50 scanpaths, indicating that doctors typically concluded their diagnosis within this range. This length is an order of magnitude larger than that of state-of-the-art gaze modeling in natural images^15^. In the supplementary material, we include the distribution plot showing the most common fixation sequence lengths.

**Table 1:** Representing the number of train/test samples for each dataset.

Dataset	Total samples	Train samples	Test samples
REFLACX	2507	1800	707
EGD-CXR	1072	800	271
REFLACX + EGD-CXR	3578	2506	1078

###  Statistical metrics

We assessed our model using two categories of metrics: fixation heatmap-based and scanpath similarity-based evaluations. For fixation heatmaps, we employ intersection over union (IoU) and correlation coefficient (CC)^30^. IoU quantifies the percentage overlap between the target and prediction masks, while CC gauges the correlation between normalized predicted and human fixation maps. Regarding scanpath similarity, we utilize the mean multimatch match score (MM)^31,32^, which aggregates scores for shape, direction, length, position, and duration. Additionally, we present the mD-MM (mean duration multimatch score), representing the duration aspect of the MM score and indicating the accuracy of fixation duration predictions. we provide 95% confidence intervals derived from the bootstrap method to ensure the robustness of our findings. For the analysis of case complexity, we compute the Pearson correlation coefficient for true and predicted total fixation durations and the spearman rank correlation coefficient for clinical workload ranks. All statistical calculations were performed using the Scikit-learn package (version 1.2.1) in Python v3.8.

## Results

Our results section is organized into three distinct parts: (1) comparison with state-of-the-art scanpath prediction methods, (2) application to clinical workload prediction, and (3) radiologist assessment of the realism of the predicted scanpaths.

### Comparison with the state of the art

**Fig. 3:**
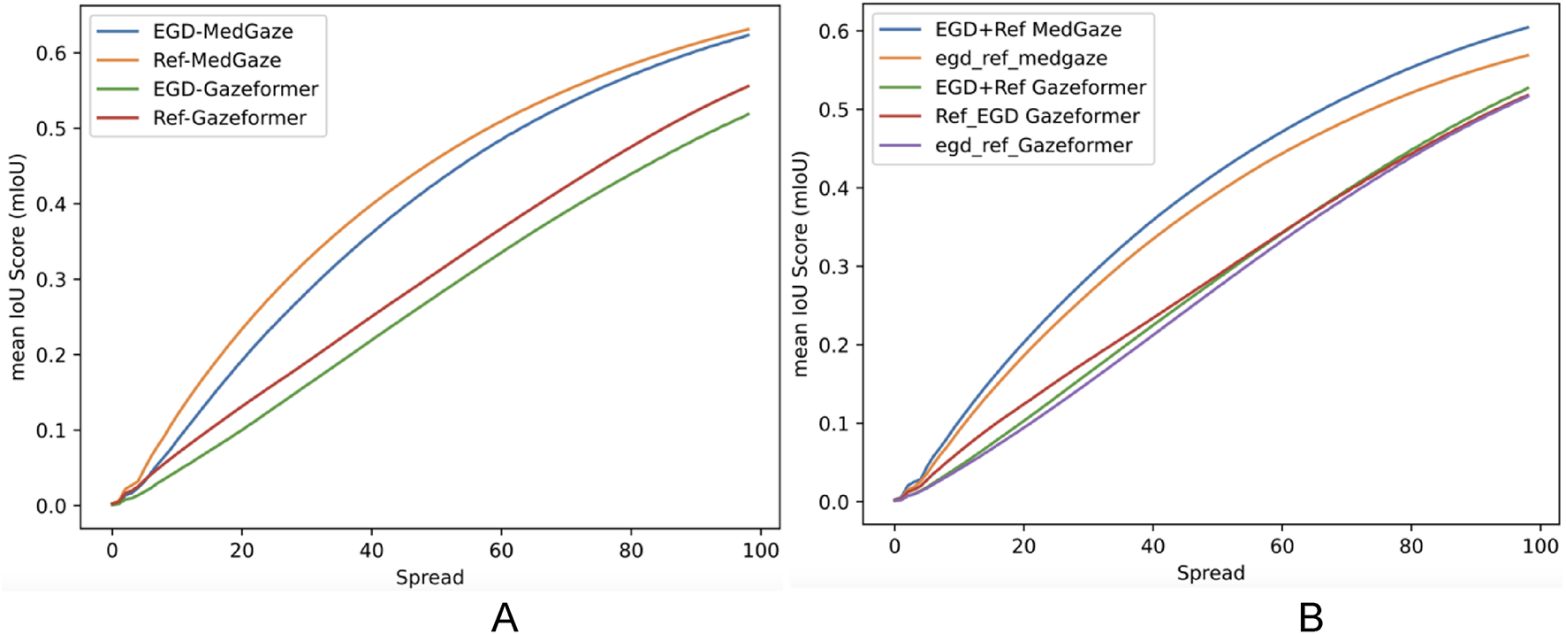
Figure represents two subplots (**A**,**B**) Subplot A depicts the IoU comparison between MedGaze and the Gazeformer across different spread levels for models trained and tested on the same dataset. Subplot B illustrates the IoU comparison between MedGaze and the Gazeformer across different spread levels for models trained and tested on different datasets.

It is essential to highlight that static fixation heatmaps are generated based on predicted fixation coordinates and fixation duration for each case. The intensity around each fixation coordinate is adjusted by scaling it with the fixation duration. For Table 2, we set the intensity spread around each fixation coordinate to 50. However, we also evaluated performance across all pixel spread levels and provided the comparison in Fig. 3.

**Table 2:** Performance comparison of medgaze and gazeformer on EGD-CXR (single experienced radiologist data) and REFLACX (multiple radiologists data).

Method	Train Dataset	Test Dataset	mIoU	mCC	mMM	mD-MM
Gazeformer^15^	EGD-CXR	EGD-CXR	0.27 (0.26, 0.28)	0.37 (0.36, 0.41)	0.71 (0.70, 0.72)	0.06 (0.04, 0.08)
	REFLACX	REFLACX	0.30 (0.29, 0.30)	0.40 (0.38, 0.42)	0.76 (0.75, 0.77)	0.29 (0.27, 0.33)
MedGaze (Ours)	EGD-CXR	EGD-CXR	0.41 (0.40, 0.421)	0.50 (0.48,0.5)	0.80 (0.79, 0.81)	0.50 (0.46, 0.52)
	REFLACX	REFLACX	0.45 (0.44, 0.46)	0.53 (0.50, 0.55)	0.84 (0.83, 0.85)	0.66 (0.65, 0.68)
Gazeformer^15^	EGD-CXR	REFLACX	0.26 (0.25, 0.27)	0.33 (0.31, 0.34)	0.69 (0.68, 0.70)	0.07 (0.05, 0.08)
	REFLACX	EGD-CXR	0.28 (0.27, 0.29)	0.38 (0.36, 0.41)	0.72(0.71,0.73)	0.19( 0.17, 0.20)
MedGaze (Ours)	EGD-CXR	REFLACX	0.39 (0.38, 0.40)	0.42 (0.40, 0.43)	0.78 (0.77, 0.79)	0.49 (0.46, 0.52)
	REFLACX	EGD-CXR	0.41 (0.40, 0.43)	0.50 (0.47, 0.51)	0.81(0.80,0.82)	0.63( 0.62, 0.64)
Gazeformer^15^	EGD-CXR + REFLACX	EGD-CXR + REFLACX	0.30 (0.29, 0.31)	0.42 (0.40, 0.43)	0.78 (0.77, 0.79)	0.43 (0.41, 0.45)
MedGaze (Ours)	EGD-CXR + REFLACX	EGD-CXR + REFLACX	0.41 (0.40, 0.42)	0.49 (0.48, 0.51)	0.85 (0.84, 0.86)	0.73 (0.72, 0.74

Values in the bracket represent the 95% confidence interval calculated using the bootstrap method.

As shown in Table 2, when trained and tested on the same dataset (same radiologist), MedGaze shows significant improvements over Gazeformer^15^. Specifically, for the EGD-CXR dataset, MedGaze achieves a mIoU of 0.41 [95% CI 0.40,0.42 ], mCC of 0.50 [95% CI 0.48,0.51 ], mMM of 0.80 [95% CI 0.79,0.81 ], and mD-MM of 0.50 [95% CI 0.46,0.52 ], compared to Gazeformer’s 0.27 [95% CI 0.26,0.28 ], 0.37 [95% CI 0.36,0.41 ], 0.71 [95% CI 0.70,0.71 ], and 0.06 [95% CI 0.048, 0.0839], respectively. On the REFLACX dataset, MedGaze achieves a mIoU of 0.45 [95% CI 0.44,0.46 ], mCC of 0.53 [95% CI 0.50,0.55 ], mMM of 0.84 [95% CI 0.83,0.85 ], and mD-MM of 0.66 [95% CI 0.65,0.68 ], while Gazeformer achieves 0.30 [95% CI 0.29,0.30 ], 0.40 [95% CI 0.38,0.42 ], 0.76 [95% CI 0.75,0.77 ], and 0.29 [95% CI 0.27,0.33 ], respectively. This substantial performance gain highlights MedGaze’s superior ability to predict radiologists’ scanpaths and fixation durations accurately.

Additionally, we assessed performance based on dataset transferability to understand how well the model generalizes across different datasets. Since the EGD-CXR and REFLACX datasets are recorded by different radiologists, it is crucial to comprehend how well the model identifies abnormal regions corresponding to text, rather than solely overfitting to a specific dataset. When trained on EGD-CXR and tested on REFLACX, MedGaze achieves a mIoU of 0.39 [95% CI 0.38, 0.40] and an mCC of 0.42 [95% CI 0.40, 0.43], outperforming Gazeformer, which scores 0.26 [95% CI 0.25, 0.27] and 0.33 [95% CI 0.31, 0.34], respectively. Conversely, when trained on REFLACX and tested on EGD-CXR, MedGaze scores 0.41 (95% CI 0.40, 0.43) for mIoU, 0.50 (95% CI 0.47, 0.51) for mCC surpassing Gazeformer’s 0.28 (95% CI 0.27, 0.29), 0.38 (95% CI 0.36, 0.41) respectively.

We also trained and tested our proposed model on the combined REFLACX + EGD-CXR dataset to evaluate whether a larger dataset would enhance scanpath prediction. Our model achieved a mIoU of 0.41 [95% CI 0.40,0.42] , mCC of 0.49 [95% CI 0.48, 0.51], mMM of 0.85 [95% CI 0.84, 0.86], and mD-MM of 0.73 [95% CI 0.72, 0.74], significantly surpassing Gazeformer’s scores of 0.30 [95% CI 0.29, 0.31], 0.42 [95% CI 0.40, 0.43], 0.78 [95% CI 0.77, 0.79], and 0.43 [95% CI 0.41, 0.45], respectively.

In Fig. 3A, it is evident that MedGaze outperforms Gazeformer across all spread levels when both models are trained and tested on the same radiologist’s data. In Fig. 3B, MedGaze also surpasses Gazeformer across all spread levels when trained and tested on different radiologists’ eye gaze datasets. Notably, the blue curve, representing MedGaze trained on a combination of both EGD-CXR and REFLACX, consistently outperforms all other curves. The orange curve, slightly below, represents MedGaze trained on EGD-CXR and tested on REFLACX. The difference between these curves (EGD_REF_MedGaze and EGD + Ref MedGaze) is more pronounced than the difference between the curves representing EGD + REF_Gazeformer and REF_EGD_Gazeformer. This indicates that MedGaze exhibits greater effectiveness and generalization when data augmentation is performed.

## Predicted scanpaths visualization

**Fig. 4:**
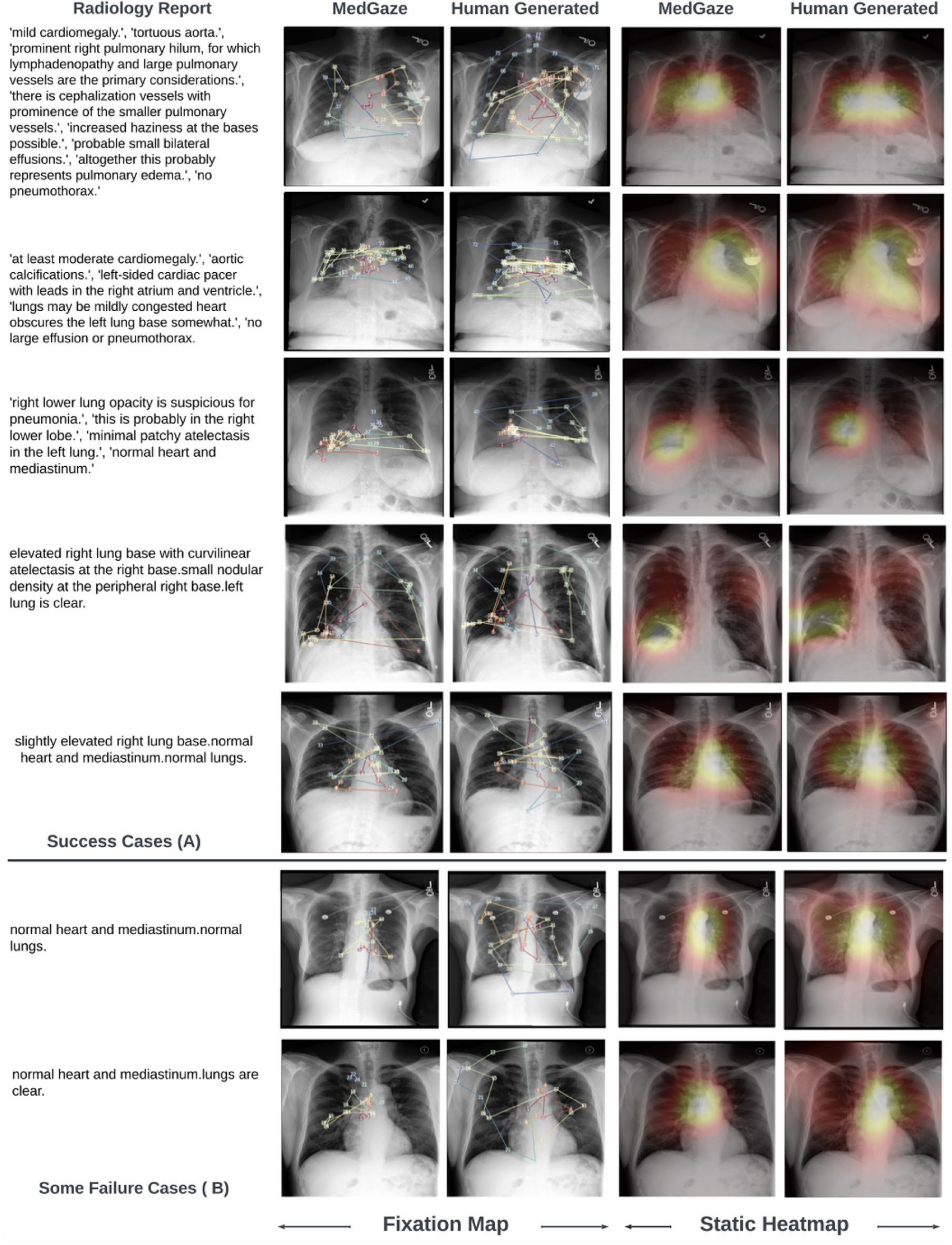
Illustration of predicted and ground truth scanpaths, scaled by fixation duration, alongside corresponding static fixation heatmaps, divided into two parts. (**A**) shows successful cases where the model’s predicted scanpaths align with the ground truth, while (**B**) highlights failure cases where the model struggles. The first column in each part presents the radiology report, while the second and third columns display the predicted and ground truth fixation coordinates, respectively. In these columns, the red arrow represents the start of the scan, and the blue arrow indicates the end. The fourth and fifth columns show the predicted and ground truth static fixation heatmaps for the entire report.

Figure 4 presents examples of predicted scanpaths from the test set of the EGD-CXR dataset, divided into two parts: Fig. 4(A) highlights successful cases where the model closely follows the ground truth scanpaths, while Fig. 4(B) illustrates failure cases where the model struggles. As previously mentioned, we set the maximum scanpath length to 50, which is significantly longer than Gazeformer’s scanpath length for natural images. However, in some cases, the actual ground truth scanpaths exceed this limit, particularly when radiologists continue examining the chest CXR beyond the initial diagnosis. Despite this limitation, our model effectively captures their post-interpretation gaze behavior within the predefined scanpath length. The model primarily focuses on predicting scanpaths corresponding to critical regions of interest rather than covering the entire image. In some instances, the ground truth scanpaths appear dispersed across various regions, making them harder to replicate precisely. Nonetheless, in several key examples, our model demonstrates strong alignment with radiologists’fixation patterns.

For example, in the third row of Fig. 4(A), where the radiologist notes, “Right lower lung opacity is suspicious for pneumonia,” the model accurately predicts fixation points around the right lower lung, where the increased opacity is observed. In the second row, where the radiologist identifies cardiomegaly, the model predicts fixation points around the heart region, even beginning the scanpath at the heart, which aligns well with the report. In the fourth row, where the radiologist diagnoses a nodular density in the right lung base, the model correctly predicts fixation points in the right lung base and follows a logical fixation sequence, starting at the heart region before shifting to the abnormality. The model also performs adequately in a normal case, shown in the fifth row, where the radiologist mentions the left lung base but reports no abnormalities. While the model predicts fixation points around the left lung base, the overall fixation pattern does not closely resemble the ground truth, likely due to the absence of specific anatomical details in the report, which makes it harder for the model to focus on relevant regions compared to abnormal cases. These successful cases demonstrate that MedGaze can accurately predict fixation points corresponding to abnormalities described in radiology reports.

However, as shown in Fig. 4(B), the model struggles in certain scenarios, particularly with some normal cases where the radiology report only mentions a normal heart and normal lungs without providing much detail about the anatomy of the CXR image. This lack of specific focus makes it harder for the model to predict accurate scanpaths. In normal cases, search patterns can vary significantly between radiologists and often lack a consistent structure. Unlike abnormal cases, which tend to focus on specific regions, normal cases typically involve scanning the entire image with more scattered scanpath patterns. This variability and broader exploration make it difficult for the model to replicate the radiologist’s gaze trajectory. Additionally, when reports indicate a normal heart or lungs, the model sometimes fails to generate diverse scanpaths that match the more dispersed ground truth patterns.

## Analyzing the clinical workload based on the fixation duration

**Fig. 5:**
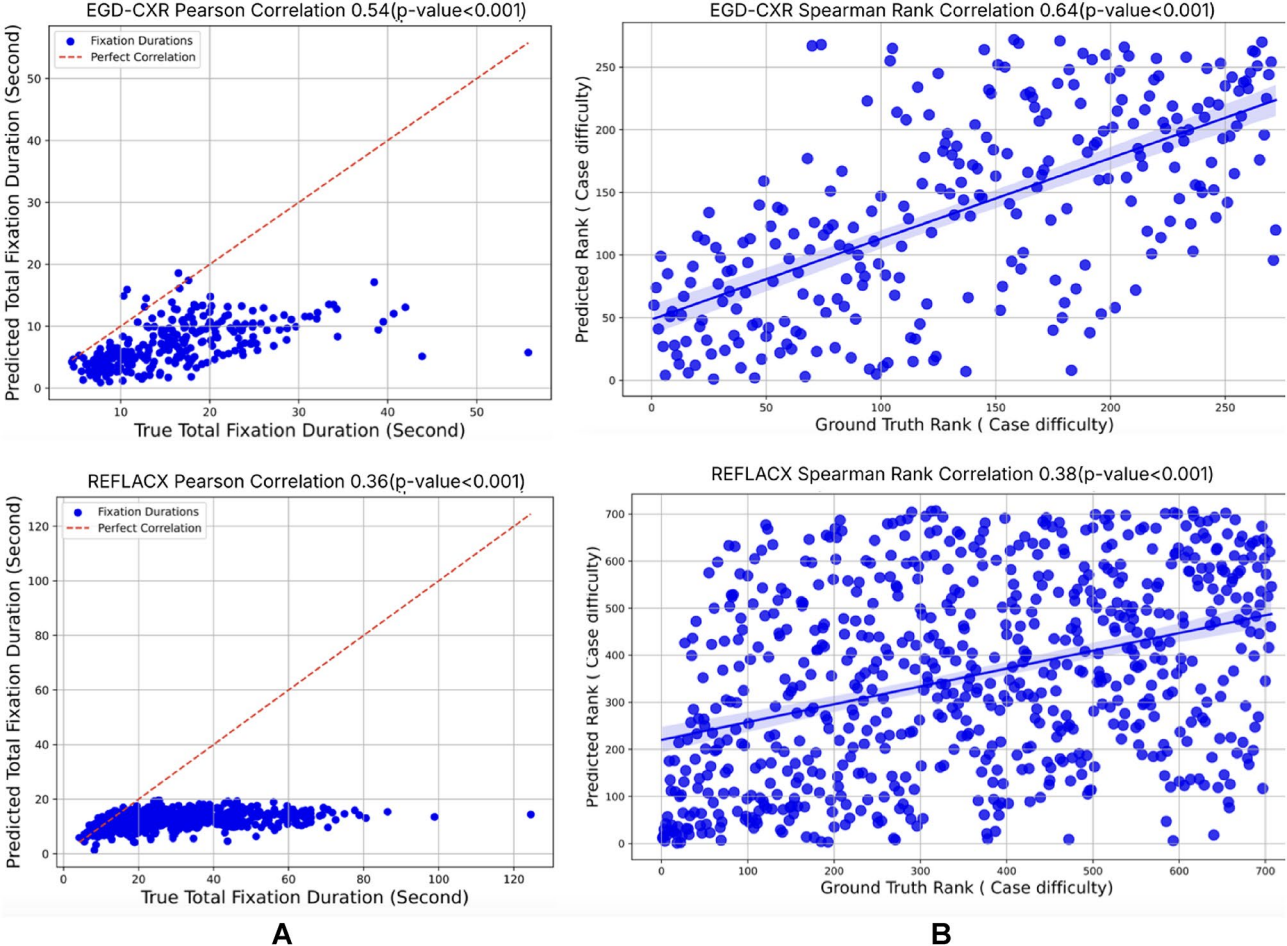
Clinical workload analysis using the Correlation coefficients. The figure comprises two columns (**A**,**B**). Column A represents the Pearson Correlation Coefficient between true and predicted total fixation duration for the EGD-CXR and REFLACX test sets. Column B represents the Spearman Rank Correlation between the true and predicted clinical workload ranks on the EGD-CXR and REFLACX test set.

Our investigation into clinical workload, inferred from fixation duration, reveals insightful findings regarding the model’s comprehension of clinical workload. When trained and tested on the EGD-CXR dataset with recordings from a single expert radiologist, the Pearson correlation coefficient (CC) between the radiologist’s time duration and the model’s predicted time duration was 0.54 (*p* < 0.001), as illustrated in Fig. 5, Column (1) Conversely, the REFLACX dataset, with recordings from five radiologists of varying experience levels, yielded a lower CC of 0.36 (*p* < 0.001). To further assess clinical workload, we ranked cases based on total predicted fixation durations, with longer durations indicating higher clinical workload (longer visual attention), in line with previous studies suggesting that longer fixation durations generally reflect increased cognitive load, task difficulty, and mental effort^33–35^. We then plotted these ranks against the ground truth ranks (based on actual fixation points and durations available in the dataset). Furthermore, for the EGD-CXR dataset, a significant positive correlation between predicted and ground ranks was evident, with a Spearman rank correlation coefficient of 0.64 (*p* < 0.001), depicted in Fig. 5, Column (2) In the REFLACX dataset, the analysis showed a lower Spearman rank correlation coefficient of 0.38 (*p* < 0.001), likely due to the dataset’s inherent noise from multiple radiologists with varying expertise levels.

**Fig. 6:**
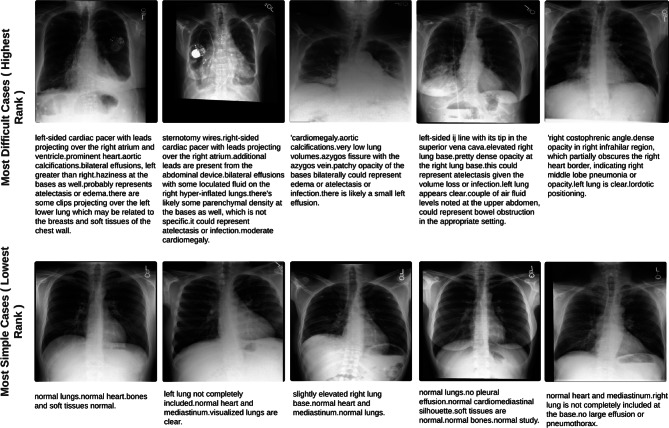
Examples from the EGD-CXR dataset test set, illustrating the most challenging and simplest cases based on their rank correlations from Fig. 4, Column 2. The first two rows show chest X-ray (CXR) images and corresponding radiology reports for cases ranked highest (most clinical workload), characterized by multiple abnormalities, and requiring longer attention. The third and fourth rows display CXR images and reports for cases ranked lowest (least clinical workload), typically showing no abnormalities or normal findings.

Figure 6 illustrates cases from the EGD-CXR test set positioned at both extremes (lowest and highest) of the distribution shown in Fig. 5, Column 2, which represents the rank correlation. The cases ranked highest, indicating the highest clinical workload scenarios, typically feature multiple abnormalities. In contrast, cases ranked lowest, denoting the lowest clinical workload scenarios, frequently involve no abnormalities or represent normal cases.

### Human radiologist evaluation

We conducted a randomized control study where a board-certified radiologist was asked to score scanpaths without the knowledge of whether they are from human radiologists or MedGaze. The results, as shown in Table 3, indicate MedGaze’s strong alignment with human gaze patterns. For identifying machine-generated versus human gaze patterns, MedGaze’s predictions were rated as human-like in 13 out of 20 instances by a radiologist, compared to 19 for the ground truth, demonstrating a high degree of human-likeness. In terms of comprehensiveness, MedGaze showed robust coverage of important regions, with 8 predictions scoring a 4 (61–80% coverage) and 10 achieving a 5 (81–100% coverage), closely matching the ground truth, which had 8 and 12 predictions in these categories, respectively. Furthermore, MedGaze exhibited minimal redundancy, with most scores at 1 or 2, indicating efficient coverage with less overlap compared to human patterns, which had more instances of moderate redundancy. Overall, MedGaze effectively mimics the human gaze while maintaining efficiency and thorough coverage of significant regions. We also provide the randomly selected 40 video and expert radiologist evaluations in the source code repository.

**Table 3:** Human evaluation of medgaze predictions compared to Human-Generated scanpaths on CXR images across defined metrics.

Criteria	Rating Scale	Prediction	Ground truth
Identifying Machine-Generated vs. Human Gaze Patterns	0: (Machine-Generated)	7	1
	1: (Human-Like)	13	19
Comprehensive Scores: Coverage of Important Regions	1: (00–20%) Very little coverage	0	0
	2: (21–40%) Some regions covered	0	0
	3: (41–60%) Fair amount of coverage	2	0
	4: (61–80%) Most regions covered	8	8
	5: (81–100%) All regions covered	10	12
Redundancy Score: Coverage of Redundant Regions	1: Minimal redundancy	9	5
	2: Some minor redundancy	7	11
	3: Moderate redundancy	3	4
	4: Significant redundancy	1	0
	5: High redundancy and inefficiency	0	0

## Discussion

This study introduces MedGaze, a novel system designed to model the complex cognitive processes of radiologists when interpreting chest CXR images. MedGaze employs a two-stage training strategy: Vision-Language Representation Learning and Vision Cognitive Learning. Initially, MedGaze is pre-trained on the publicly available MIMIC dataset to learn medically relevant multimodal features. Subsequently, the pre-trained MedGaze undergoes end-to-end training with the EGD-CXR and REFLACX datasets, aiming to predict scanpaths over CXR images. Our system is thoroughly evaluated using statistical metrics and human evaluation.

Table 2 presents a performance comparison between MedGaze and the state-of-the-art (SOTA) method Gazeformer across different train/test combinations. Notably, when trained and tested on the same datasets (either EGD-CXR or REFLACX), MedGaze consistently outperforms Gazeformer in all metrics: mIoU, mCC, mMM, and mD-MM. For instance, on the REFLACX dataset, MedGaze achieves an mIoU of 0.45 [95% CI 0.44, 0.46], an mCC of 0.53 [95% CI 0.50, 0.55], an mMM of 0.84 [95% CI 0.83, 0.85], and an mD-MM of 0.66 [95% CI 0.65, 0.68], significantly higher than Gazeformer’s 0.30 [95% CI 0.29, 0.30], 0.40 [95% CI 0.38, 0.42], 0.76 [95% CI 0.75, 0.77], and 0.29 [95% CI 0.27, 0.33], respectively. The substantial difference in mD-MM scores for both datasets, EGD-CXR (MedGaze: 0.50 [95% CI 0.46, 0.52] vs. Gazeformer: 0.06 [95% CI 0.048, 0.0839]) and REFLACX (MedGaze: 0.66 [95% CI 0.65, 0.68] vs. Gazeformer: 0.29 [95% CI 0.27, 0.33]), highlights MedGaze’s superior ability to predict fixation duration, crucial for understanding clinical workload. This performance can be attributed to our two-stage training approach, which effectively captures the intricate visual attention patterns of radiologists.

Our results also highlight the impact of dataset size on model performance. Both MedGaze and Gazeformer exhibit enhanced performance when trained on the larger REFLACX dataset compared to the smaller EGD-CXR dataset. This discrepancy is particularly evident in the metrics, with MedGaze’s performance on REFLACX (mMM of 0.84 [95% CI 0.83, 0.85]) surpassing that on EGD-CXR (mMM of 0.80 [95% CI 0.79, 0.81]). This finding underscores the importance of large, diverse training datasets in improving model accuracy and generalizability.

Another crucial aspect of our study is MedGaze’s ability to generalize across different radiologists. When trained on one dataset and tested on another (e.g., trained on REFLACX and tested on EGD-CXR), MedGaze still demonstrates robust performance, albeit with a slight decrease compared to training and testing on the same dataset. For example, MedGaze’s mIoU drops from 0.45 (95% CI 0.44, 0.46) when trained and tested on REFLACX to 0.41 [95% CI 0.40, 0.43] when trained on REFLACX and tested on EGD-CXR.

To further validate MedGaze’s effectiveness, we created a larger dataset by combining the REFLACX and EGD-CXR datasets. MedGaze achieved an mIoU of 0.41 [95% CI 0.40, 0.42], an mCC of 0.49 [95% CI 0.48, 0.51], an mMM of 0.85 [95% CI 0.84, 0.86], and an mD-MM of 0.73 [95% CI 0.72, 0.74], significantly higher than Gazeformer’s scores of 0.30 [95% CI 0.29, 0.31], 0.42 [95% CI 0.40, 0.43], 0.78 [95% CI 0.77, 0.79], and 0.43 [95% CI 0.41, 0.45], respectively. Although there is a slight decrease in performance when combining data from different radiologists compared to training and testing on the same radiologist’s data, our model still showed good performance. This suggests that combining data from multiple radiologists acts as a regularizer, introducing noise into the training process and aiding in generalizing the model across multiple datasets.

Radiologists’ interpretation of CXRs entails varying fixation durations, influenced by multiple factors such as the complexity of findings, the number of abnormalities present, and their level of expertise, etc. To evaluate a model’s ability to grasp clinical workload, we analyzed the Pearson correlation coefficient between predicted and ground truth total fixation durations. When tested on the EGD-CXR dataset (collected on experienced radiologist), the model demonstrated a significant positive correlation (CC = 0.54, *p* < 0.001), indicating its tendency to predict longer durations for challenging cases However, when tested on the REFLACX dataset, which includes recordings from radiologists with varying levels of expertise, the correlation coefficient decreased (CC = 0.36, *p* < 0.001). This lower correlation highlights the increased noise in the dataset, resulting from differences in the radiologists’ experience and expertise. Each radiologist has their own unique search strategy, which can vary significantly based on their familiarity with the case, cognitive load, and level of proficiency. For instance, less experienced radiologists may spend more time exploring normal regions due to uncertainty, while experts exhibit more efficient and targeted gaze patterns. This variation in fixation patterns introduces complexity for the model, making it difficult to discern consistent and generalized patterns of clinical workload. Additionally, external factors such as fatigue, prior exposure to similar cases, and cognitive biases further influence how long radiologists focus on certain areas, contributing to inconsistencies in gaze behavior. These factors amplify the noise in the dataset, which reduces the model’s ability to accurately predict fixation durations and, as a result, the clinical workload.

Expanding our investigation, we ranked clinical workload based on total fixation duration, with longer fixation durations corresponding to higher clinical workload, thus representing higher ranks. For the EGD-CXR dataset, a significant positive correlation between predicted and ground truth ranks was evident, with a Spearmanrank correlation coefficient of 0.64 (*p* < 0.001). The rank-order plot revealed a clear trend, particularly for cases with longer fixation durations. However, in the REFLACX dataset, the Spearman correlation was lower (0.38, *p* < 0.001), due to dataset noise arising from diverse expertise levels among radiologists. To better understand workload differences, we plotted the cases with the highest and lowest clinical workload (highest rank). Most cases with the highest clinical workload typically exhibit multiple abnormalities requiring careful attention for accurate diagnosis, whereas the simplest cases often depict normal conditions without abnormalities. This ranking approach can effectively guide the development of training programs for novice radiologists by presenting cases in increasing order of difficulty. Beginning with straightforward cases( normal cases with no abnormality) allows beginners to grasp normal anatomy and basic abnormalities, progressing to more challenging cases with longer fixation durations to refine their skills in identifying subtle or atypical findings. Such structured training enhances diagnostic accuracy and confidence, equipping radiologists to effectively manage diverse clinical scenarios while fostering continuous professional development.

The evaluation of MedGaze using human-likeness and comprehensiveness criteria reveals insightful findings about its performance in predicting gaze patterns. MedGaze’s predictions were rated as human-like in 13 out of 20 cases, compared to 19 out of 20 for the ground truth, indicating a high degree of accuracy in emulating human gaze behavior. In terms of comprehensiveness, MedGaze demonstrated strong coverage of important regions, with 8 predictions scoring a 4 (61–80% coverage) and 10 predictions achieving a perfect score of 5 (81–100% coverage). This performance is comparable to the ground truth, where 8 and 12 predictions scored 4 and 5, respectively. However, the redundancy scores suggest that MedGaze predictions are less redundant than human gaze patterns, with a majority of its scores falling between 1 and 2 (minimal to some minor redundancy), while human patterns had more instances of moderate redundancy. This indicates that MedGaze not only effectively identifies crucial regions but also does so more efficiently, avoiding unnecessary fixation on redundant areas. Overall, the results underscore MedGaze’s ability to closely mimic human gaze patterns while enhancing efficiency in gaze prediction.

Despite the promising results, several limitations must be acknowledged. The datasets used (REFLACX and EGD-CXR) are limited in size and diversity, which could affect the model’s generalizability. The eye-tracking data, derived from a small number of radiologists, may not fully capture the complexity of human visual behavior, another limitation is the model’s performance with some normal cases. While it excels in cases where radiologists focus on specific abnormalities, it struggles with certain normal cases, particularly when reports lack detailed anatomical descriptions like “normal heart” or “normal lungs.” In these cases, radiologists’ search patterns are more exploratory and variable, lacking a consistent structure. This variability makes it challenging for the model to replicate the diverse gaze behaviors of radiologists, resulting in less accurate predictions.Additionally, MedGaze currently focuses solely on chest X-rays, and its applicability to other medical imaging modalities, such as CT and MRI, remains unexplored. The computational cost and complexity of large multimodal models could also hinder real-time clinical deployment.

## Conclusion

In conclusion, MedGaze represents a significant advancement in predicting scanpaths on chest X-ray (CXR) images by modeling the cognitive processes of radiologists through a two-stage training framework. Leveraging large publicly available datasets, MedGaze outperforms the state-of-the-art Gazeformer in fixation coordinate and duration prediction, demonstrating superior IoU, CC, and Multimatch scores across diverse datasets. Its robust generalizability was validated through both quantitative metrics and human evaluations. These results suggest that MedGaze holds potential for enhancing training programs for novice radiologists and optimizing clinical workflows. While the current study emphasizes the technical development of an AI model for predicting gaze sequences in radiology reports, we see MedGaze as a promising component for future training systems. Beyond its technical contributions, MedGaze aligns with broader research on expertise development and medical education. Gaze patterns are valuable indicators of expertise, but expertise acquisition also involves structured cognitive strategies such as deliberate practice and guided reflection^36^. Future work will explore the integration of MedGaze with deliberate practice frameworks^37^, incorporating structured reasoning exercises and adaptive feedback mechanisms to refine diagnostic decision-making. Additionally, we aim to expand dataset diversity, extend MedGaze’s applicability to other imaging modalities, and integrate it into real-time radiology training systems. By bridging AI-driven gaze prediction with cognitive training strategies, MedGaze has the potential to advance both research and practical applications in medical image interpretation.

## Electronic supplementary material

Below is the link to the electronic supplementary material.

Supplementary Material1

## Data Availability

Data availability: We have used the public datasets for our study. Links for the dataset are below: REFLACX (https://physionet.org/content/reflacx-xray-localization/1.0.0/) EGD-CXR (https://physionet.org/content/egd-cxr/1.0.0/).

## Code availability

We provide the pre-trained model and dataset in this link (https://github.com/a04101999/MedGaze).
