# Supplementary material for: Modeling radiologists’ cognitive processes using a digital gaze twin to enhance radiology training
Source: Sci Rep. 2025 Apr 21;15:13685. doi: 10.1038/s41598-025-97935-y (PMC12012060; doi:10.1038/s41598-025-97935-y)
Supplement: Supplementary file 1 — Supplementary Material1 [file 41598_2025_97935_MOESM1_ESM.pdf]

Supplementary Information

Ablation Experiments:

Before finalizing the architecture outlined above, we conducted numerous ablation experiments to test various configurations. A notable series of experiments focused on modeling within the multimodal space, exploring different combinations of the Medformer and Large Language Models (LLM). Furthermore, we experimented with replacing the visual backbone using the Clip-based Vision Transformer (ViT). However, this change led to increased computational costs while delivering performance comparable to the current visual backbone, prompting us to stick with the simpler model. Table 4 illustrates the diverse combinations of ablation experiments and their respective comparisons. We also ventured into data augmentation on the EGD-CXR dataset by generating multiple permutations or views of each data point. This process involved rearranging the text in the radiology reports and adjusting the corresponding eye gaze sequences accordingly. However, these experiments displayed relatively poor performance when combining both the EGD-CXR and REFLACX datasets, indicating that adding different views of the same data points resulted in modal collapse.

**Category**	**Method/Configuration**	**IoU**
Training strategy	Single training(Qformer)	0.25
Training strategy	Dual Training(MedFormer)	0.30
Multimodal space	LLM	0.31
Multimodal space	MedFormer+LLM	0.42
Visual Backbone	Resnet50 + Transformer	0.42
Visual Backbone	Clip ViT	0.40
Data Augmentation	Permutation based	0.34
Data Augmentation	Real data-based	0.41

Table S1: Ablation table: Comparison of diverse ablation experiments exploring training strategies, multimodal modeling, and visual backbone variations, highlighting the performance outcomes and insights gained.

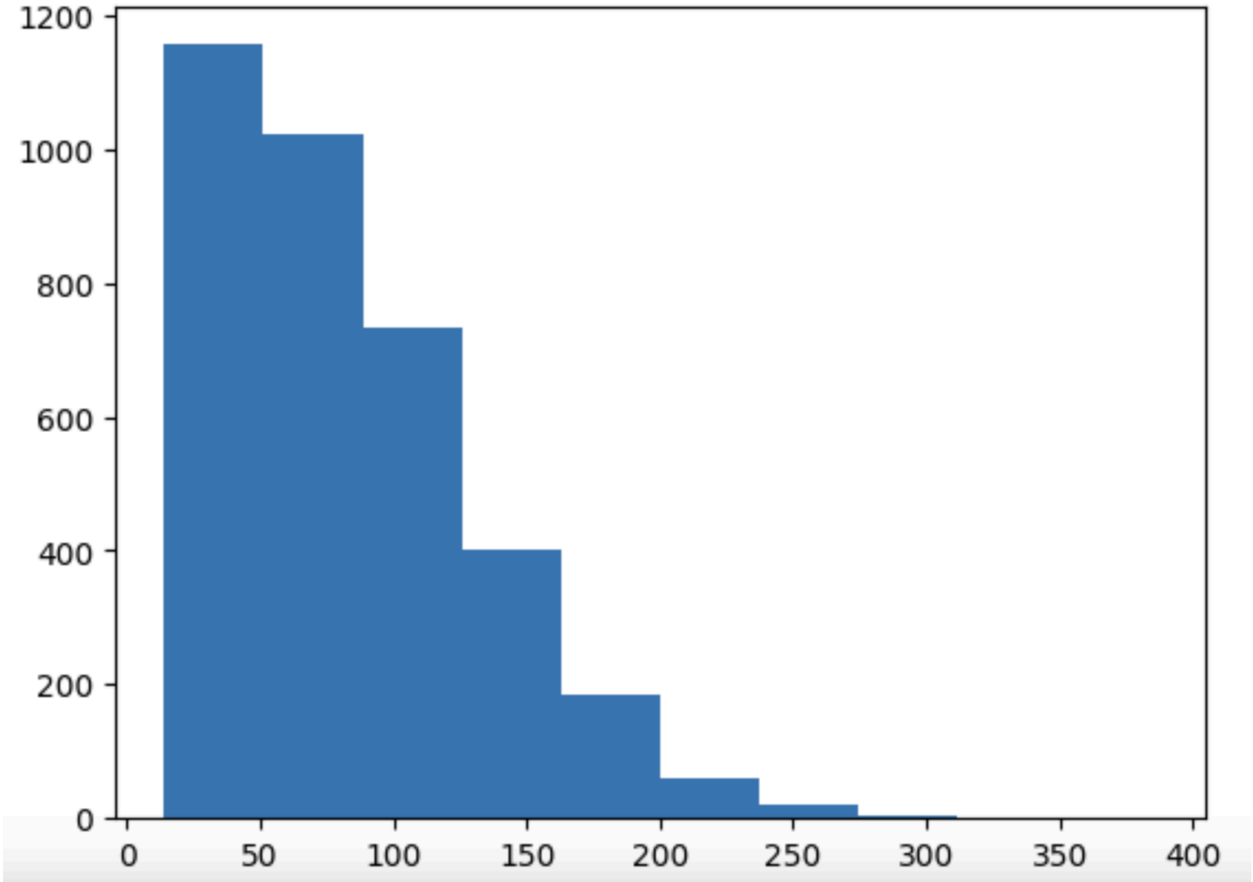

Figure S1: Histogram showing the median Fixation length in the eye gaze data is about 50
